# Sotos Syndrome Is Associated with Deregulation of the MAPK/ERK-Signaling Pathway

**DOI:** 10.1371/journal.pone.0049229

**Published:** 2012-11-14

**Authors:** Remco Visser, Ellie B. M. Landman, Jelle Goeman, Jan M. Wit, Marcel Karperien

**Affiliations:** 1 Department of Pediatrics, Leiden University Medical Center, Leiden, The Netherlands; 2 Department of Developmental BioEngineering, MIRA Institute for Biomedical Technology and Technical Medicine, Enschede, The Netherlands; 3 Department of Medical Statistics and Bioinformatics, Leiden University, Medical Center, Leiden, The Netherlands; University of California, San Francisco, United States of America

## Abstract

Sotos syndrome (SoS) is characterized by tall stature, characteristic craniofacial features and mental retardation. It is caused by haploinsufficiency of the *NSD1* gene. In this study, our objective was to identify downstream effectors of NSD1 and to map these effectors in signaling pathways associated with growth. Genome-wide expression studies were performed on dermal fibroblasts from SoS patients with a confirmed *NSD1* abnormality. To substantiate those results, phosphorylation, siRNA and transfection experiments were performed. A significant association was demonstrated with the Mitogen-Activated Protein Kinase (MAPK) pathway. Members of the fibroblast growth factor family such as *FGF4* and *FGF13* contributed strongly to the differential expression in this pathway. In addition, a diminished activity state of the MAPK/ERK pathway was demonstrated in SoS. The Ras Interacting Protein 1 (RASIP1) was identified to exhibit upregulated expression in SoS. It was shown that RASIP1 dose-dependently potentiated bFGF induced expression of the MAPK responsive SBE reporter providing further support for a link between NSD1 and the MAPK/ERK signaling pathway. Additionally, we demonstrated *NSD1* expression in the terminally differentiated hypertrophic chondrocytes of normal human epiphyseal growth plates. In short stature syndromes such as hypochondroplasia and Noonan syndrome, the activation level of the FGF-MAPK/ERK-pathway in epiphyseal growth plates is a determining factor for statural growth. In analogy, we propose that deregulation of the MAPK/ERK pathway in SoS results in altered hypertrophic differentiation of *NSD1* expressing chondrocytes and may be a determining factor in statural overgrowth and accelerated skeletal maturation in SoS.

## Introduction

Sotos syndrome (SoS; MIM 117550) is characterized by tall stature, facial dysmorphism and mental retardation [1]. It is caused by haploinsufficiency of the nuclear receptor binding SET domain protein 1 (*NSD1)* gene [2]. Originally, mouse Nsd1 was identified in a two-hybrid screen with the retinoic acid receptor alpha (RARα) as bait [3]. It was shown that Nsd1 interacted with a number of nuclear hormone receptors, such as the estrogen receptor, retinoic acid and thyroid hormone receptors [3]. It was postulated that NSD1 could either act as a co-repressor or a co-activator of these nuclear receptors depending on the cellular context and the presence or absence of the respective hormones [3]. Since then, several *in vitro* experiments showed that an important function of NSD1 is the methyltransferase activity of its Su(var)3–9, Enhancer-of-zeste and Trithorax (SET) domain [4]–[6]. This domain specifically methylates lysine 36 at histone H3 (H3-K36), lysine 20 at histone H4 (H4-K20) and other non-histone substrates (4-7), resulting overall in the regulation of chromatin transcription. For example, NSD1 was shown to regulate activity of the transcription factor NF-κB [7]. Furthermore, depletion of NSD1 reduced the expression of the bone morphogenetic protein gene 4 (*BMP4*) [5]. In addition, a fusion protein NUP98–NSD1 which is found in about 5% of human acute myeloid leukemia patients, was demonstrated to regulate transcription of the Hox-A gene locus [8]. In a tissue-specific manner NSD1 exhibits both repressing as well as activating capacities as was exemplified by reduced expression of the *MEIS1* oncogene in a neuroblastoma model, while increased expression was detected in transfected cells containing the NUP98–NSD1 fusion protein [8], [9].

Based on its role in regulation of gene transcription, it has been hypothesized that heterozygous inactivation of NSD1 results in loss of repression of growth promoting genes [4]. Consequently, increased activity of these genes would lead to the characteristic overgrowth phenotype of SoS. Unfortunately, experimental evidence for this hypothesis is lacking due to the fact that heterozygous knock out mice of Nsd1 do not show a SoS phenotype [4], especially with regard to overgrowth and epiphyseal growth plate changes (prof. Losson, personal communication).

Our group previously studied the expression of members of the Growth Hormone (GH)/Insulin like growth factor 1 (IGF1) axis in SoS patients [10]. Modestly increased plasma levels of IGFBP-2 and IGFBP-6 and reduced levels of IGF-I, IGF-II, IGFBP-3 and IGFBP-4 were detected. Reduced levels of IGF-I and IGF-II in particular are, however, more reminiscent of short rather than tall stature such as observed in SoS. The relationship between NSD1 and the GH/IGF1 axis remains elusive. Thus, although a number of functions of NSD1 have been identified, the molecular mechanisms leading to phenotypic features of SoS remain largely unclear.

The aim of the current study was therefore to identify downstream effectors of NSD1 and to map these effectors in signaling pathways associated with growth. Genome-wide mRNA expression profiles of dermal fibroblasts from SoS patients with a confirmed *NSD1* abnormality were compared to expression patterns in age and sex-matched controls.

## Materials and Methods

### Ethics Statement

This study was approved by the Medical Ethical Committee of the Leiden University Medical Center and written informed consent was given by the patients and/or their parents or legal guardians.

### Subjects

The study included cell lines of skin fibroblasts which were obtained from 9 SoS patients with a confirmed *NSD1* abnormality and 9 sex- and age matched normal donors as described previously by De Boer *et al.* [10]. Details are presented in Tables S1 [11] and S2.

### Cell Culture

Skin fibroblasts were cultured in Dulbecco’s modified Eagle’s Medium supplemented with glutamax (Gibco, Breda, The Netherlands) and containing 10% fetal calf serum (Lonza, Verviers, Belgium). The cell cultures were maintained at 37°C in a humidified atmosphere of 5% CO_2_ and 95% O_2_. In all experiments cell passages were between 2 and 10. With regard to the NSD1 interaction with RARα [3], we hypothesized that differences in gene expression between fibroblasts of SoS patients and controls would be more pronounced in the presence of all trans-retinoic acid (RA). At reaching 80–90% monolayer confluence cells were therefore stimulated with vehicle or with RA (Sigma-Aldrich, St Louis, MO, USA) at a final concentration of 10^−6^ M for 48 h. After this period, cells were still viable and proliferating. Because of the light-sensitivity of RA, cell cultures were kept in the dark.

### Total RNA Extraction, Labeling and Microarray Hybridization

After total RNA extraction and concentration measurement, quality and integrity were checked and biotin-labeling was performed by ServiceXS Leiden according to manufacturer’s guidelines (Affymetrix, Santa Clara, CA, USA). Hybridization to GeneChip HG-U133 Plus 2.0 Arrays (Affymetrix) and scanning was conducted by the Leiden Genome Technology Center following manufacturer’s protocols. The whole genome HG-U133 Plus 2.0 Array contains 54120 probe sets covering 38572 UniGene clusters.

### Statistical Analysis

The microarray expression data was deposited at the NCBI Gene Expression Omnibus database (accession number GSE27200). Analyses of the microarray data were made in R (version 2.7.0; www.r-project.org). Probe level data was preprocessed with the Robust Multi-array Average (RMA) algorithm [12] in the Bioconductor affy package (version 1.18.2) [13]. Analyses of differentially expressed genes were performed using the Linear Models for Microarray Data (limma, version 2.14.5) package [14]. A correction for multiple testing was performed using the Benjamin-Hochberg method.

The Global Test package (version 4.10.0) was used for the pathway analysis [15]. Details about the pathways can be found in Table S3. The permutation method was employed and correction for multiple testing was performed. In all statistical analysis, results with an adjusted p.value<0.05 were considered statistically significant.

### Real-Time Quantitative PCR

Real-Time quantitative PCR (qPCR) using QuantiTect primers (Qiagen, Venlo, The Netherlands) was performed to validate differentially expressed genes identified in the microarray analysis. The following genes were selected: *RASIP1*, *RBM47*, *COMP, and FGF13*. Fold changes were adjusted for the expression of the housekeeping gene β2 microglobulin following the 2^−ΔΔCt^ method [16].

### NSD1 siRNA Study

HEK293t cells (American Type Culture Collection CRL-11268) were cultured for 48 hours after which cells were transfected with siRNA (Qiagen) for *NSD1* or non-targeting siRNA and incubated for another 48 hours. After RNA isolation, expression levels were determined with qPCR.

### RASIP1 Transfection Study

HEK293t cells (American Type Culture Collection CRL-11268) were co-transfected with the MAPK-responsive SBE promoter reporter construct in combination with increasing amounts of a *RASIP1* expression vector and pRL-CMV Renilla control, using Fugene HD (Roche Applied Science, Almere, The Netherlands). pUC19 DNA was used to adjust DNA concentrations in the transfection experiment. Twenty-four hours after transfection, cells were stimulated with 10 ng/ml bFGF for 24 hours. Luciferase values were normalized for Renilla and expressed as fold induction compared to control which was set to 1+/− standard error of mean. For statistical analysis, OneWay ANOVA was performed.

### Protein Phosphorylation Studies

Cells were cultured for 48 h in full medium and subsequently RA was added with a final concentration of 10^−6^ M. Cells were then incubated for another 48 h. This was followed by incubation in serum free DMEM containing 0.1% bovine serum albumin (Sigma-Aldrich, St. Louis, USA) for 14 hrs. Cells were lysed and protein concentrations were subsequently diluted to 250 µg/ml. Phosphorylated and total ATF2, cJun, ERK1/ERK2, ERK2, HSP27, JNK, MEK1, p38MAPK and p90RSK were detected using the Bio-Plex suspension array system (www.bio-rad.com). This method for the detection of multiple phosphorylated and non-phosphorylated proteins was shown previously to have a good correlation with Western blots [17]–[19], but in contrast less protein is needed for multiplex analysis. Quantitative differences in phosphorylation were examined with a correction for total protein levels by univariate analysis. P-values of <0.05 were considered significant.

### Immunohistochemistry

Two monoclonal anti-NSD1 antibodies (1NW1A10 and 3NW3F8) recognizing distinct NSD1 epitopes were obtained with kind permission from prof. dr. R. Losson at IGBMC/GIE-CERBM, France [3]. Immunohistochemistry was performed as previously described [20]. Negative controls included the omission of the first antibody. Human growth plates included were of different developmental ages: fetal (17 weeks), 1 year and 13 years of age. Details about the growth plates can be found in the legend of Figure S3.

## Results

### Differentially Expressed Genes in Sotos Syndrome

To study differences in gene expression profiles in SoS, RNA obtained from dermal fibroblasts from nine SoS patients with confirmed *NSD1* alterations was compared with dermal fibroblast RNA from nine age and sex matched controls using genome wide expression profiling. The significant probe sets and their corresponding genes are shown in Table 1 and 2. In basal situation, five probe sets were differentially expressed after correction for multiple testing. These probes sets corresponded to 4 genes: *Ras i*nteracting *p*rotein *1* (*RASIP1)*, Plakophilin 3 (*PKP3)*, RNA binding motif 47 (*RBM47)*,and *KIAA0895*. After RA stimulation, the differential expression of *RASIP1* and *RBM47* was preserved and 2 new genes (Mucolipin 3 (*MCOLN3)* and *KIAA1128)* were identified to be differentially expressed. Significance of the PKP3 and KIAA0895 probe sets was lost. Expression of *RASIP1* and *PKP3* was up regulated in SoS, while expression of the other identified genes was down regulated.

**Table 1:** Differentially expressed probe sets in SoS in basal situation.

Nr	Probe set ID	Gene symbol	Gene name	Entrez ID	^2^Log Fold Change	p.value	p.value adjusted for multiple testing
1	220027_s_at	RASIP1	Ras interacting protein 1	54922	2.1	1.75E–08	0.001
2	209873_s_at	PKP3	plakophilin 3	11187	0.4	4.87E–07	0.013
3	222496_s_at	RBM47	RNA binding motif protein 47	54502	−0.5	1.43E–06	0.026
4	213424_at	KIAA0895	KIAA0895 protein	23366	−0.4	2.87E–06	0.033
5	218035_s_at	RBM47	RNA binding motif protein 47	54502	−0.5	3.00E–06	0.033

**Table 2:** Differentially expressed probe sets in SoS after stimulation with RA.

Nr	Probe set ID	Gene symbol	Gene name	Entrez ID	^2^Log FoldChange	p.value	p.value adjusted for multiple testing
1	220027_s_at	RASIP1	Ras interacting protein 1	54922	2.0	2.36E–07	0.013
2	229797_at	MCOLN3	mucolipin 3	55283	−0.7	6.79E–07	0.019
3	222496_s_at	RBM47	RNA-binding motif protein 47	54502	−0.8	1.44E–06	0.026
4	240499_at	KIAA1128	KIAA1128	54462	−0.7	2.93E–06	0.040

qPCR was performed for *RASIP1*, *RBM47* and two additional controls: *Fibroblast Growth Factor 13* (*FGF13*) as an example of the most down regulated gene in SoS (fold change of 13.5; p = 0.18) and *COMP* (fold change 4.8; p = 0.15) gene as an example of an up regulated gene. For all genes, the qPCR results confirmed the gene expression profiles detected with the microarray (Figure S1), although for *RASIP1* considerably higher fold changes (16.1 vs 4.1) were found with qPCR.

### Differentially Expressed Signaling Pathways in Sotos Syndrome

To study the association of NSD1 with signal transduction pathways, 10 KEGG signaling pathways and 26 GO-terms were selected based on a previously established role in growth regulation and/or NSD1 function [3], [4]. A global test was performed independently from the Limma-analysis and included all probe sets of the whole genome expression microarrays, both in basal or RA stimulated condition (Table 3). In the basal situation none of the signaling pathways was significantly associated with SoS. After stimulation with RA, a significant association was detected with the mitogen activated protein kinase (MAPK) pathway (adjusted p.value = 0.023). This was further supported by the significant association (adjusted p-value = 0.003) with the MAPK kinase kinase cascade GO-term, which partially overlaps in gene content with the KEGG MAPK pathway. Furthermore, although no significant associations with other KEGG signaling pathways were found after correction for multiple testing, we noted that stimulation with RA improved adjusted p-values for all pathways compared to the basal situation (Table 3).

**Table 3:** Signaling pathways analysis results.

		Basal situation		RA stimulation	
KEGG pathway number	Name	p.value	FDR adj.p.value	p.value	FDR adj. p.value
4010	MAPK signaling pathway	0.062	0.341	0.002	0.023
4012	ErbB signaling pathway	0.090	0.341	0.020	0.087
4310	Wnt signaling pathway	0.102	0.341	0.026	0.087
4330	Notch signaling pathway	0.218	0.546	0.036	0.089
4350	TGF-beta signaling pathway	0.324	0.648	0.074	0.148
4370	VEGF signaling pathway	0.516	0.677	0.138	0.230
4630	Jak-STAT signaling pathway	0.550	0.677	0.192	0.274
4020	Calcium signaling pathway	0.556	0.677	0.247	0.309
4070	Phosphatidylinositol signaling system	0.610	0.677	0.299	0.332
4150	mTOR signaling pathway	0.870	0.870	0.758	0.758
**GO-term**	**Name**				
0000165	MAPKKK cascade	0.003	0.082	1.23E–04	0.003

**Figure 1:**
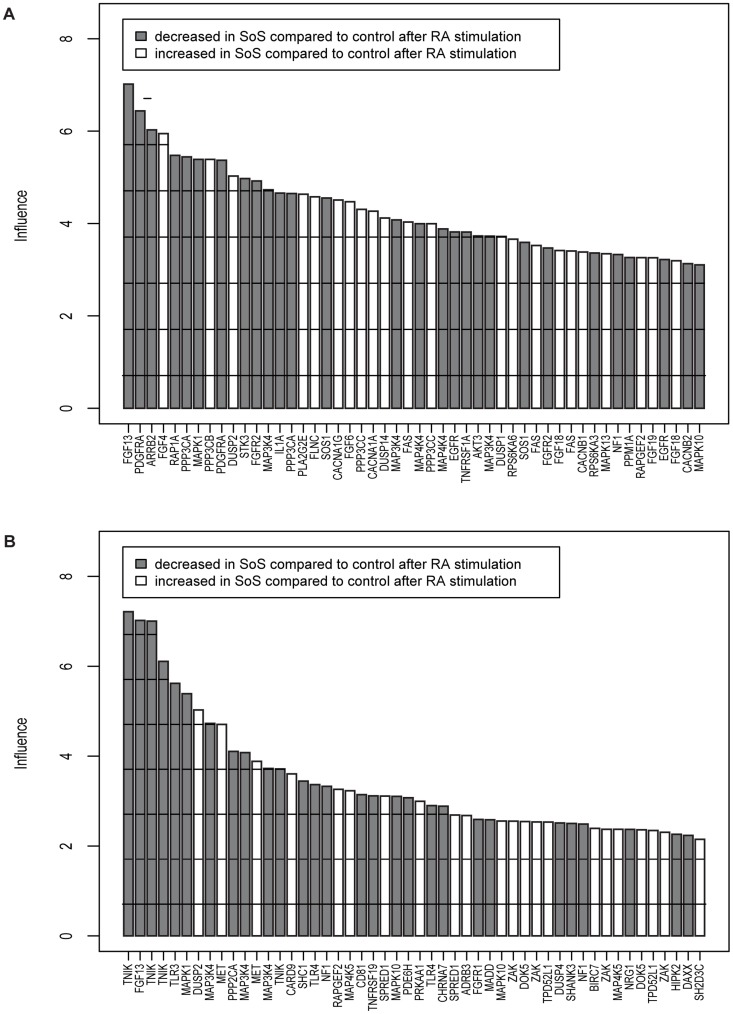
Geneplots of the probe sets influencing the MAPK pathway. Geneplots are shown for the 50 most influential probe sets from the KEGG MAPK pathway (A) and for the GO-term MAPKKK cascade (B) after stimulation with RA that contribute to the differential pathway expression in SoS and control. Probe sets are scaled to unit standard deviations and the height of the bars are the number of standard deviations above the cut-off level of 0.7. Higher bars indicate higher influence on the pathway. Probe sets with the highest influence on the pathway (i.e. FGF13 in Figure 1A and TNIK in Figure 1B) are depicted on the left. Corresponding gene names are written below each bar.

The KEGG MAPK pathway consists of 781 probe sets and the GO MAPKKK cascade contains 433 probe sets. We determined the contribution of each of these probe sets to the differential expression of the MAPK pathway in SoS versus controls after RA treatment. The 50 most influential probe sets are shown in Figure 1. The probe set for *FGF13* contributed strongly to the differential expression of the MAPK pathway and MAPKKK cascade. *FGF13* was the most down regulated gene in SoS after RA-treatment (fold change 13.5), although this down regulation did not reach significance after correction for multiple testing (p = 0.18). Besides *FGF13* also *FGF4*, *FGF6*, *FGF18*, *FGF19* and the *FGFR2* contributed strongly to the differential expression of the MAPK pathway. The signature of the FGF-signaling pathway as observed in the KEGG pathway analysis was less clear in the GO MAPKKK pathway analysis.

### The Most Differentially Expressed Gene between SoS and Controls is RASIP1

**Figure 2:**
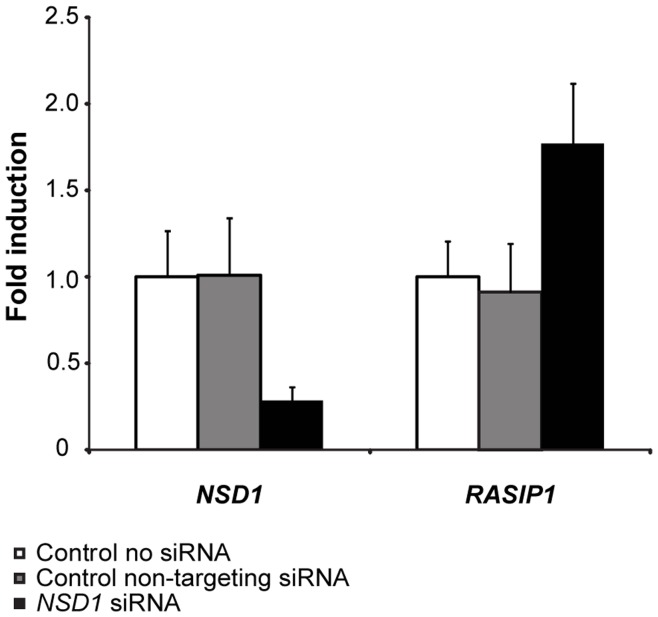
*NSD1* knock down increases *RASIP1* expression. Expression of *NSD1* was downregulated with siRNA (black bar on the left), which resulted after 48-hours in up regulation of *RASIP1* expression (black bar on the right), while no difference in expression was detected for the controls (white and grey bars). Fold change represents the average difference in expression level of the respective gene. Fold changes were adjusted for the expression of the housekeeping gene β2 microglobulin using the 2^−ΔΔCt^ method.

**Figure 3:**
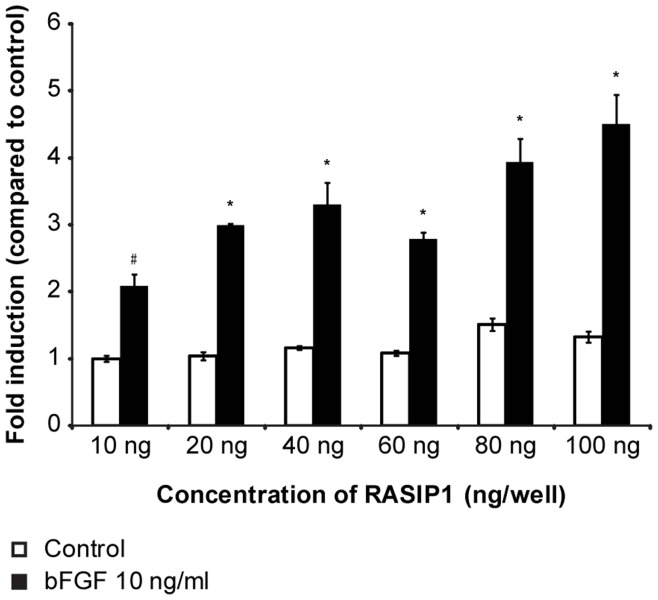
bFGF induced SBE reporter activation is potentiated by RASIP1. Values are expressed as fold induction compared to control. Cells were stimulated with bFGF for 24 hours. Control was not stimulated with bFGF and no RASIP1 was co-transfected. Co-tranfection of RASIP1 did not affect basal reporter activity. bFGF (10 ng/ml) significantly stimulated SBE reporter activity (indicated with #; p<0.05). RASIP1 enhanced bFGF induced reporter dose-dependently (indicated with *; p<0.05).

RASIP1 specifically interacts with GTP-bound Ras and acts as an effector of endomembrane localized Ras [21], which plays an important role in MAPK-signaling. To study the role of RASIP1 in MAPK signaling we showed that a siRNA induced knock down of *NSD1* results in up regulation of *RASIP1* mRNA expression (Figure 2). This is in line with the microarray data and confirms that *RASIP1* expression is regulated by the expression levels of *NSD1*. As shown in Figure 3, transient transfection of a RASIP1 expression vector increased MAPK-luciferase reporter activity dose-dependently, particularly in the presence of bFGF. As RASIP1 is not included in the KEGG and GO-terms, these results provide an independent line of evidence of altered MAPK-signaling in SoS.

### Differential Activation of the MAPK Pathway in SoS

**Figure 4:**
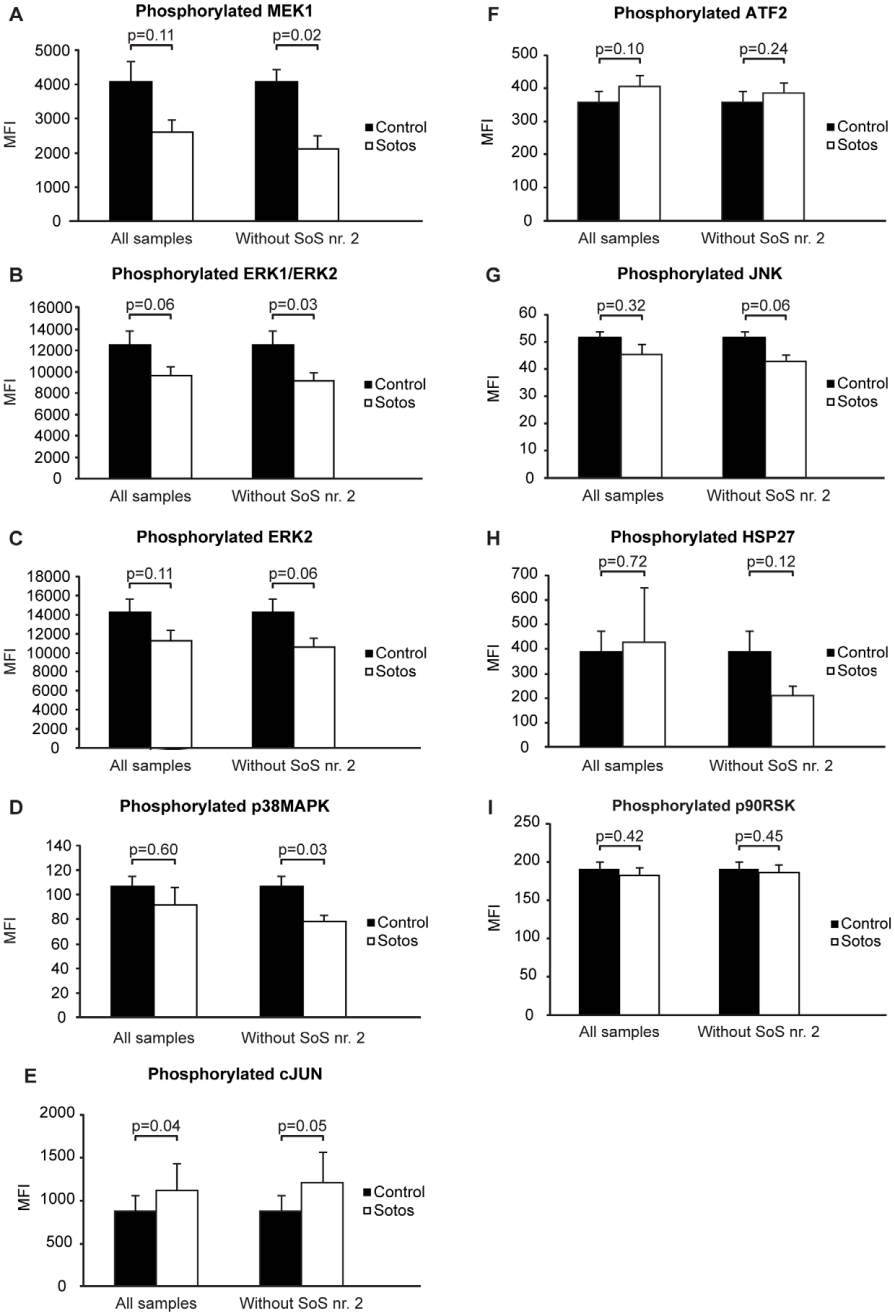
Protein phosphorylation. The results are shown for the phosphorylation levels for the MEK1 (A), ERK1/ERK2 (B), ERK2 (C), p38MAPK (D), cJUN (E), ATF2 (F), JNK (G), HSP27 (H) and p90RSK kinases (I). Bar heights depict the mean fluorescence intensity levels measured (MFI) and the p-values for the difference between SoS and control (after correction for total protein levels) are shown above the bars.

We next determined whether the differential expression of the MAPK pathway in SoS cell lines after RA stimulation was corroborated with differential activation of this pathway at the protein level. We focused on determining the activation status of key kinases (ERK1/ERK2, ERK2, JNK, MEK1, p38MAPK and p90RSK) of this pathway and downstream effectors like the transcription factors (ATF2, cJUN) and the heath shock protein HSP27 in basal condition and after stimulation with RA. A trend was observed for a lower activation state of the kinases MEK1, ERK1/ERK2, and ERK2 in basal conditions, but this did not reach significance (p = 0.06–p = 0.11) (Figure 4).

Critical assessment of all data points of phosphorylated proteins revealed, however, that the phosphorylation level of a number of proteins in fibroblasts of SoS patient nr. 2 were remarkably higher than the values observed in other SoS fibroblasts as well as in the control group. These differences could not be explained by experimental variation in e.g. protein loading. Principal component analysis including all data points of phosphorylated proteins, clearly demonstrated that the response in this cell line was distinct from other SoS and control fibroblast cultures (Figure S2). This was further confirmed using the outlier detection method in the simple and the sophisticated sign method both indicated the patient as an outlier at a critical value of 0.99 [22].

Analysis was repeated after removal of the fibroblasts of patient no. 2 from the SoS group. Now the activation state of MEK1, ERK1/ERK2 and p38MAPK in basal conditions was significantly reduced in SoS (Figure 4). The basal activity of ERK2 was also reduced but this did not reach significance (p = 0.06). Removal of the outlier did not change the activation state observed for the other proteins. In RA stimulated cells, the activation state of MEK1 (p = 0.02) was lower in SoS, while no differences were observed between the other proteins.

### NSD1 is Expressed in the Human Growth Plate

Longitudinal growth is regulated by a complex interplay of multiple growth factors and their receptors in the epiphyseal growth plate [23]. We employed immunohistochemistry to study the expression of NSD1 in human growth plate specimens. NSD1 was expressed in the terminally differentiated hypertrophic chondrocytes at different developmental ages (Figure S3).

## Discussion

In order to elucidate biological pathways explaining how NSD1 haploinsufficiency results in phenotypic features such as overgrowth in SoS a comprehensive study of dermal fibroblasts from SoS patients was performed. We obtained evidence that SoS syndrome is associated with a deregulation of the MAPK/ERK signaling pathway. The MAPK/ERK signaling pathway is an important regulator of cell differentiation, proliferation and apoptosis [24]. More recently, activating mutations in this pathway have been identified as the causative factor in a number of short stature syndromes, such as Noonan syndrome and Costello syndrome [25].

Deregulated MAPK/ERK signaling pathway in SoS is based on the following observations. First, a significant association of *NSD1* expression and the MAPK/ERK signaling pathway was shown in the fibroblast microarray study. Second, *RASIP1*, a downstream Ras effector and hence interfering with the RAS/MAPK/ERK signaling cascade, was observed to be upregulated in SoS. Knock down experiments confirmed *RASIP1* as a NSD1 target. Third, lower phosphorylation levels in SoS of several key MAP kinases of the MAPK/ERK pathway were detected. Fourth, in a transfection model, *RASIP1* dose-dependently potentiated bFGF induced expression of the MAPK- responsive SBE reporter construct. One may argue that the associations in some of the individual experiments were not strong or revealed a more complex picture (e.g. the results of the phosphorylation experiments). However the analysis of each independently performed experiment points into the same direction and when taken together they provide strong evidence for a deregulation of the MAPK/ERK signaling pathway in SoS.

Microarray analysis identified a relatively small number of significantly differentially expressed genes. This is likely explained by the relatively small number of samples, due to the limited number of SoS patients available, in combination with a considerable level of biological variation, hence reducing the power of the experiment to obtain significance [26]. In addition, dermal fibroblasts may not be the most optimal model for studying the effects of NSD1 haploinsufficiency in relation to overgrowth. However dermal fibroblasts do express *NSD1* [27] and dermal fibroblasts have successfully been used to elucidate molecular mechanisms underlying growth disorders [28]–[31]. Furthermore, in marked contrast to growth plate chondrocytes they are easily available for analysis. Additionally, to our knowledge there are no existing humane chondrocyte cell lines derived from growth plate chondrocytes yet available. We therefore believe that dermal fibroblasts are suited for studying the molecular mechanisms underlying the overgrowth in SoS. Because 8 of 9 Sotos syndrome patients harboured a single nucleotide mutation which did not affect mRNA stability (Table S1), altered expression of NSD1 was not detected in the array experiments.

The first line of evidence linking NSD1 to deregulated MAPK/ERK signalling was derived from the pathway analysis. Given the established role of NSD1 in RA signalling, we hypothesized that the effects on gene expression and pathway analysis between SoS and control would be more pronounced after treatment with RA. Indeed, differences in gene expression and pathway analysis became more pronounced after RA treatment in support of our hypothesis. This might be due to an increase in fold change of the genes in the MAPK pathway after RA treatment. However such an effect was not seen in the phosphorylation studies. An explanation for this difference might be the 48 hour period of stimulation with RA, which is sufficient to detect differences on an mRNA level, but may be too short to reflect differences at a protein level.

The second independent line of evidence for an association with the MAPK/ERK kinase signaling pathway was derived from the Limma analysis, which identified *RASIP1* as the number 1 differentially expressed gene between SoS and control. Knock down experiments confirmed RASIP1 as a direct target of NSD1. RASIP1 interacts in a GTP-dependent manner with endomembrane-associated Ras and is recruited to the Golgi by activated Ras (Ras-GTP) [21]. RASIP1 binds to several members of the Ras family and is proposed to be a downstream Ras-effector, which is a central player in the MAPK/ERK pathway [21]. The physiological significance of such compartmentalized signaling of Ras, i.e. Ras signaling on endosomes such as endoplasmic reticulum and the Golgi apparatus, has not been elucidated yet [32]. Since it was shown that Golgi-associated Ras was able to activate the MAPK signaling cascade in a timely and quantitatively different manner, it was proposed that compartmentalized Ras signaling would increase the complexity of possibilities for downstream signaling [33]. Therefore, increased RASIP1 expression in interference with activated Ras, might be related to the differences in cell growth and differentiation observed in SoS.

Third line of evidence is the trend of lower phosphorylation levels of MEK1, ERK1/ERK2, ERK2 in SoS in basal conditions and for MEK1 after stimulation with RA. This trend became significant after exclusion of SoS nr. 2 as an outlier. Lower phosphorylation levels of key kinases would indicate a decreased activation of the MAPK/ERK pathway in SoS. A fourth line of evidence shows that in our transfection experiments RASIP1 dose-dependently potentiated bFGF induced expression of the SBE reporter in HEK293 cells, substantiating a role for RASIP1 in the MAPK/ERK pathway. Increased MAPK/ERK pathway activity after overexpression of RASIP1 in HEK293cells seems in apparent conflict with evidence for decreased MAPK/ERK pathway activity in primary cell cultures of SoS fibroblasts. This discrepancy can be explained by the use of different cell types. Furthermore, the role of RASIP1 in compartmentalized signaling of Ras/MAPK might be more complex than can be simulated in our transfection model [30]. In addition, NSD1 was shown to influence the expression of many genes involved in the MAPK pathway (Figure 1) and the net effect of these changes might be more important for the MAPK/ERK activity state than a single up regulation of RASIP1 in a transient transfection assay.

Based on our phosphorylation date in primary cell cultures, our data point to decreased activity of the MAPK/ERK pathway in SoS.

Lower activity rather than increased activity of the pathway in SoS is also in line with literature since there is a clear association of an increased activity state of the MAPK/ERK pathway with short stature [25], [34]. The differential expression of the MAPK pathway in our microarray study was strongly dependent on differential expression of a number of FGF family members (*FGF4*, *FGF6*, *FGF13*, *FGF18*, *FGF19* and *FGFR2*). FGF signaling in the epiphyseal growth plate through the MAPK/ERK pathway is known to play an important role in skeletal development [35]. Gain-of-function alterations of the *FGFR3* gene are the cause in several well-known growth failure disorders due to impaired endochondral bone formation such as achondroplasia and hypochondroplasia [34]. Mutant mice with a constitutive active *FGFR3* mutation were dwarfed with shortened, disorganized ephiphyseal growth plates containing few proliferating and hypertrophic chondrocytes [36]. As an opposite phenotype, *Fgfr3* ^−/−^ mice showed skeletal overgrowth with increased long bones and vertebrae [37], [38]. The epiphyseal growth plates of these mice had an increased height caused by expansion of the zone of proliferating and hypertrophic chondrocytes [37], [38]. In general, FGFR3 is proposed to act as a negative regulator of bone growth by two downstream mechanisms: i.e. inhibiting chondrocyte proliferation through the STAT1-pathway [39] and by inhibiting hypertrophic chondrocyte differentiation through the MAPK-pathway [40]. Not only upstream but also downstream components of the FGF-MAPK/ERK pathway are involved in growth regulation. Constitutive active mutations in more downstream genes such as *KRAS* and *BRAF* result in an increased activation of the MAPK/ERK pathway and hence in short stature syndromes as for example Noonan or cardio-facio-cutaneous syndrome [25], [34].

All these observations suggest that the level of activation of the MAPK/ERK pathway is a determining factor for longitudinal growth and that this is regulated at the hypertrophic chondrocytes of the epiphyseal growth plate [40]. This is especially interesting, since we have shown *NSD1* expression in the terminally differentiated chondrocytes of normal human epiphyseal growth plates during different developmental ages (Figure S3). It is therefore tempting to speculate that deregulated MAPK/ERK signaling in SoS results in altered hypertrophic differentiation of *NSD1* expressing chondrocytes and that this may be a determining factor in statural overgrowth and accelerated skeletal maturation in SoS.

In conclusion, for the first time SoS is shown to be associated with deregulation of the MAPK/ERK pathway. An altered activity of this pathway may be an important contributor to the longitudinal overgrowth.

## Supporting Information

Figure S1 **qPCR validation of differentially expressed genes.** Differential gene expression of *RASIP1*, *RBM47*, *FGF13* (splice variants A and B; C represents a primer set detecting both splice-forms) and *COMP* was studied using qPCR. Fold change represents the average difference in expression level of the respective gene after stimulation with RA between the SoS-fibroblasts and controls. They were adjusted for the expression of the housekeeping gene β2 microglobulin using the 2^−ΔΔCt^ method. Black bars depict the fold changes detected with microarray and white bars show the average fold change of triplicate qPCR experiments. Fold changes indicating down regulated expression are represented with negative values. Error bars represent the standard error of the mean.
(TIF)

Figure S2 **Results of the outlier analysis.** Bi-plots of the principal component analysis on the phosphorylation levels of all investigated proteins are shown for basal condition in (A) and after stimulation with RA in (B). Circles correspond with the 9 control samples and triangles with the Sotos samples. The arrow points to the detected outlier.
(TIF)

Figure S3 **NSD1 expression in the human growth plate.** Expression of NSD1 is shown in the femoral growth plate of a fetus at the age of 17 weeks (A), in a toe of a 1 year old subject (B) and in the tibial growth plate of a 13 year old subject (C). NSD1 is expressed in the terminally differentiated hypertrophic chondrocytes. Identical immunostaining was observed for both monoclonal antibodies, which are directed against distinct epitopes of NSD1. Furthermore, immunostaining using unrelated monoclonal antibodies (i.e. extracellular matrix proteins) showed distinct staining patterns (data not shown). The fetal growth plate was obtained from the tibia of a normally developed aborted fetus. The growth plate from a one year old patient was obtained from a surgically removed 6^th^ toe in an otherwise healthy, normally growing and developing infant. The growth plate of a 13 year old patient was obtained from the femur head after surgery because of epiphyseolysis. This patient exhibited tall stature, without a specific diagnosis.
(TIF)

Table S1 **Characteristics of Sotos syndrome patients.**
(DOC)

Table S2 **Characteristics of controls.**
(DOC)

Table S3 **Analyzed KEGG signaling pathways and GO-terms.**
(DOC)
