# Supplementary figures and images for: Sotos Syndrome Is Associated with Deregulation of the MAPK/ERK-Signaling Pathway
Source: PLoS One. 2012 Nov 14;7(11):e49229. doi: 10.1371/journal.pone.0049229 (PMC3498325; doi:10.1371/journal.pone.0049229)

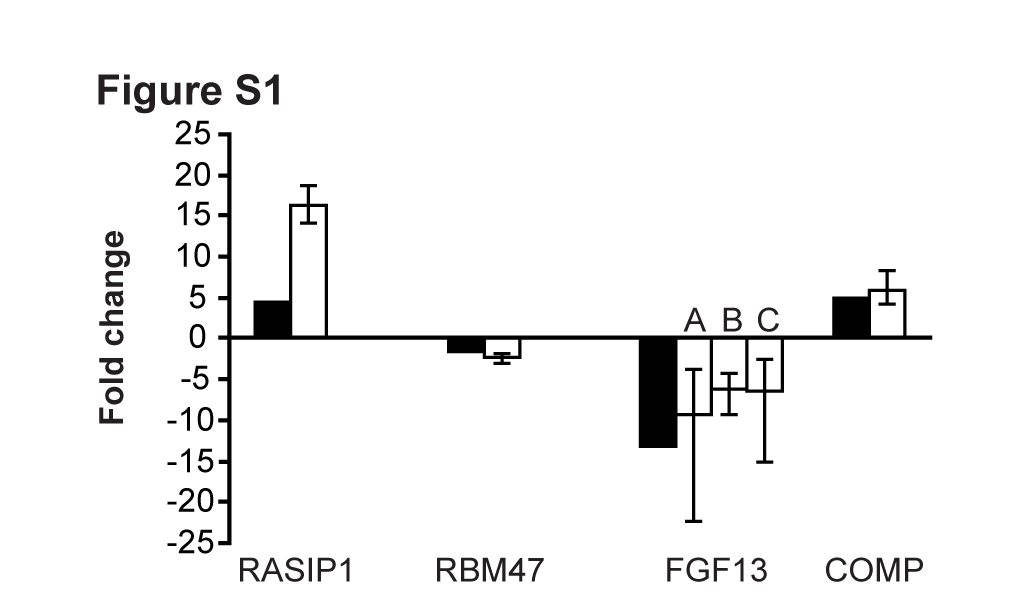

Supplement: Figure S1 — qPCR validation of differentially expressed genes. Differential gene expression of RASIP1, RBM47, FGF13 (splice variants A and B; C represents a primer set detecting both splice-forms) and COMP was studied using qPCR. Fold change represents the average difference in expression level of the respective gene after stimulation with RA between the SoS-fibroblasts and controls. They were adjusted for the expression of the housekeeping gene β2 microglobulin using the 2−ΔΔCt method. Black bars depict the fold changes detected with microarray and white bars show the average fold change of triplicate qPCR experiments. Fold changes indicating down regulated expression are represented with negative values. Error bars represent the standard error of the mean. (TIF) [file pone.0049229.s001.tif]

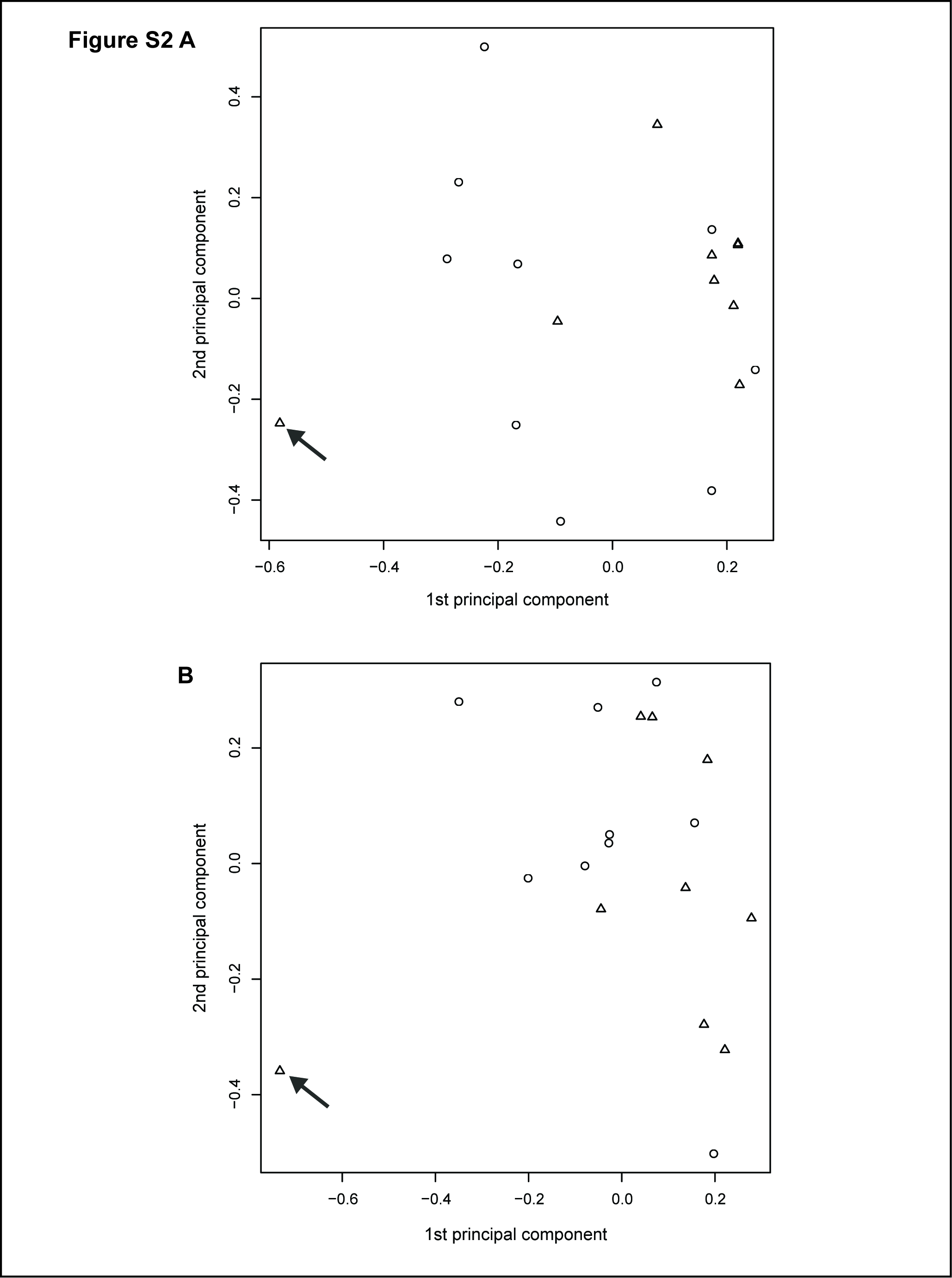

Supplement: Figure S2 — Results of the outlier analysis. Bi-plots of the principal component analysis on the phosphorylation levels of all investigated proteins are shown for basal condition in (A) and after stimulation with RA in (B). Circles correspond with the 9 control samples and triangles with the Sotos samples. The arrow points to the detected outlier. (TIF) [file pone.0049229.s002.tif]

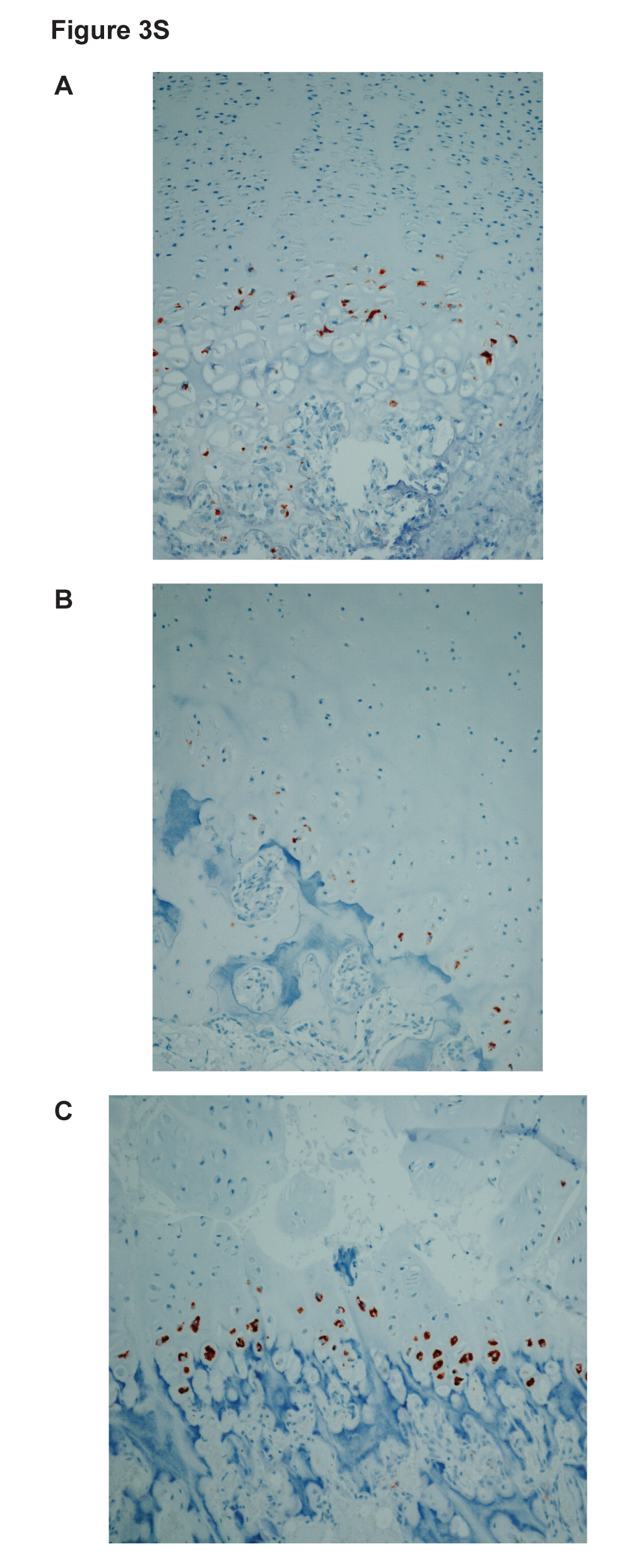

Supplement: Figure S3 — NSD1 expression in the human growth plate. Expression of NSD1 is shown in the femoral growth plate of a fetus at the age of 17 weeks (A), in a toe of a 1 year old subject (B) and in the tibial growth plate of a 13 year old subject (C). NSD1 is expressed in the terminally differentiated hypertrophic chondrocytes. Identical immunostaining was observed for both monoclonal antibodies, which are directed against distinct epitopes of NSD1. Furthermore, immunostaining using unrelated monoclonal antibodies (i.e. extracellular matrix proteins) showed distinct staining patterns (data not shown). The fetal growth plate was obtained from the tibia of a normally developed aborted fetus. The growth plate from a one year old patient was obtained from a surgically removed 6th toe in an otherwise healthy, normally growing and developing infant. The growth plate of a 13 year old patient was obtained from the femur head after surgery because of epiphyseolysis. This patient exhibited tall stature, without a specific diagnosis. (TIF) [file pone.0049229.s003.tif]
