# Supplementary material for: Sotos Syndrome Is Associated with Deregulation of the MAPK/ERK-Signaling Pathway
Source: PLoS One. 2012 Nov 14;7(11):e49229. doi: 10.1371/journal.pone.0049229 (PMC3498325; doi:10.1371/journal.pone.0049229)
Supplement: Table S1 — Characteristics of Sotos syndrome patients. (DOC) [file pone.0049229.s004.doc]

| **Table S1. Characteristics of Sotos syndrome patients.** | | | | | |
| --- | --- | --- | --- | --- | --- |
| **Number** | **Age at biopsy** | **Sex^a^** | **Nucleotide change** | **Predicted protein**  **change** | **Identifier in our previous study (11)** |
| 1 | 3.2 | M | c.6463+1G>A | p.? | Patient 10 |
| 2 | 4.5 | F | c.6241T>G | p.L2081V | Patient 28 |
| 3 | 5.7 | F | c.5950C>G | p.R1984G | Patient 11 |
| 4 | 7.4 | M | c.4548_4549delGGinsC | p.E1516fsX | Patient 2 |
| 5 | 12.5 | M | Deletion *NSD1* exon 1-3 and *FGFR4* |  | Patient 4 |
| 6 | 15.2 | M | c.5435T>A | p.V1812D | Patient 14 |
| 7 | 17.8 | M | c.2809delCGinsT | p.R937X | Patient 3 |
| 8 | 18.9 | F | c.3531delT | p.F1177fsX | Patient 8 |
| 9 | 33.5 | M | c.4108C>T | p.Q1370X | Patient 34 |

^a^ M: Male; F: Female
