# Supplementary material for: Sotos Syndrome Is Associated with Deregulation of the MAPK/ERK-Signaling Pathway
Source: PLoS One. 2012 Nov 14;7(11):e49229. doi: 10.1371/journal.pone.0049229 (PMC3498325; doi:10.1371/journal.pone.0049229)
Supplement: Table S2 — Characteristics of controls. (DOC) [file pone.0049229.s005.doc]

| **Table S2. Characteristics of controls.** | | | | |
| --- | --- | --- | --- | --- |
| **Number** | **Age at biopsy** | **Sex^a^** |  |  |
| 1 | 3.7 | F |  |  |
| 2 | 4 | F |  |  |
| 3 | 5.4 | M |  |  |
| 4 | 7 | M |  |  |
| 5 | 11.6 | M |  |  |
| 6 | 15.9 | M |  |  |
| 7 | 16.1 | M |  |  |
| 8 | 17.1 | M |  |  |
| 9 | 32 | M |  |  |

^a^ M: Male; F: Female
