# Supplementary material for: Sotos Syndrome Is Associated with Deregulation of the MAPK/ERK-Signaling Pathway
Source: PLoS One. 2012 Nov 14;7(11):e49229. doi: 10.1371/journal.pone.0049229 (PMC3498325; doi:10.1371/journal.pone.0049229)
Supplement: Table S3 — Analyzed KEGG signaling pathways and GO-terms. (DOC) [file pone.0049229.s006.doc]

| **Table S3. Analyzed KEGG signaling pathways and GO-terms^1^.** | |
| --- | --- |
| **KEGG pathway number** | **Description** |
| 4010 | MAPK signaling pathway |
| 4012 | ErbB signaling pathway |
| 4310 | Wnt signaling pathway |
| 4330 | Notch signaling pathway |
| 4350 | TGF-beta signaling pathway |
| 4370 | VEGF signaling pathway |
| 4630 | Jak-STAT signaling pathway |
| 4020 | Calcium signaling pathway |
| 4070 | Phosphatidylinositol signaling system |
| 4150 | mTOR signaling pathway |
|  |  |
| **GO-term** | **Description** |
| GO:0000165 | MAPKKK cascade |
| GO:0003713 | Transcription coactivator activity |
| GO:0004707 | MAPK activity |
| GO:0004879 | Ligand-dependent nuclear receptor activity |
| GO:0007173 | Epidermal growth factor receptor signaling pathway |
| GO:0007179 | Transforming growth factor beta receptor signaling pathway |
| GO:0007219 | Notch signaling pathway |
| GO:0007259 | JAK-STAT cascade |
| GO:0008063 | Toll signaling pathway |
| GO:0008277 | Regulation of G-protein coupled receptor protein signaling |
| GO:0016570 | Histone modification |
| GO:0016922 | Ligand dependent nuclear receptor binding |
| GO:0019722 | Calcium-mediated signaling |
| GO:0019933 | cAMP-mediated signaling |
| GO:0030111 | Regulation of Wnt receptor signaling pathway |
| GO:0030296 | Protein tyrosine kinase activator activity |
| GO:0030509 | BMP signaling pathway |
| GO:0030522 | Intracellular receptor-mediated signaling pathway |
| GO:0031929 | TOR signaling pathway |
| GO:0043550 | Regulation of lipid kinase activity |
| GO:0045814 | Negative regulation of gene expression, epigenetic |
| GO:0045815 | Positive regulation of gene expression, epigenetic |
| GO:0045892 | Negative regulation of transcription, DNA dependent |
| GO:0045893 | Positive regulation of transcription, DNA dependent |
| GO:0048010 | Vascular endothelial growth factor receptor signaling pathway |
| GO:0048011 | Nerve growth factor receptor signaling pathway |

^1^. Prior to analysis, a selection was made of 10 Kyoto Encyclopedia of Genes and Genomes (KEGG; www.genome.jp/kegg) signaling pathways and 26 Gene Ontology (GO; www.geneontology.org) terms.
